# Evaluation of a hybrid model of a Physician Associate Studies programme: students, teachers, and examiner perspectives

**DOI:** 10.1007/s11845-025-03918-3

**Published:** 2025-03-01

**Authors:** Pauline Joyce, Melanie Cunningham, Lisa Alexander

**Affiliations:** Royal College of Surgeons in Ireland (RCSI), Dublin, Ireland

**Keywords:** Connected, Hybrid, Online learning, Social isolation

## Abstract

**Background:**

To date, there is one university offering the Physician Associate (PA) Studies programme in Ireland. Responding to a demand for PAs outside of Dublin, a hybrid model was introduced, allowing students undertake the didactic phase of the programme online for synchronous instruction, attending campus twice a month for anatomy learning, clinical skills, and integration sessions.

**Aims:**

The aim of this study was to evaluate the hybrid model from the perspectives of students and their teachers, including the external examiner.

**Methods:**

This was a mixed methods study, using surveys (*n* = 15), focus groups with students (*n* = 10), teaching staff (*n* = 4), and a one-to-one interview with an external examiner. Action points of programme board meetings were also analysed.

**Results:**

While attitudes to online learning were positive, the need for improvements was highlighted. During online teaching sessions, the students expressed the need to feel more a part of the traditional classroom experience. Even though lecturers were aware of students online, some found it difficult to engage with these students. Clinical teachers did not detect any differences between hybrid and on-campus students on their clinical application to practice.

**Conclusion:**

Findings suggest that hybrid learning is influenced by previous online learning experiences. Students admitted to a feeling of social isolation at times and the reminder for faculty to support an inclusive environment. Student performance showed that hybrid students did as well or better across both years of the programme, and this translated into clinical practice too.

## Background

Since its inception, the MSc in Physician Associate Studies programme has attracted more students from Dublin county than from other parts of Ireland. Data analysed in 2022 from university records indicated that while 51% of students and alumni were born in the capital city where the programme is run, 63% resided outside the city, but within the county. Employment opportunities, however, showed an increase in areas such as the south and west of the country, due mainly to an understanding of the benefits of the PA role from consultants who have worked with PAs in North America or who have previously worked at the hospital, where the PA pilot project took place [1]. The university began piloting a hybrid model of the PA studies programme with their January 2023 intake. There were six students on the hybrid model, taking all their lectures and small group teaching online, coming to campus twice a month for anatomy and clinical teaching. Eight students attended full-time in the classroom. The number of students in the January 2024 cohort increased to 16 hybrid students and 14 on-campus. This number increase was influenced by HSE funding for tuition fees for that year, an effort to increase the number of PAs. All students were allocated to PA mentors and faculty advisors [2]. In addition, peer-to-peer meetings between on-campus and hybrid students were set up on a regular basis, in an effort to increase social interaction [3, 4]. The programme is run over two calendar years.

The hybrid model of education refers to a combination of online teaching with face-to-face and hands-on immersive education experiences [5], which has gained some traction across health professions education [6]. Since the COVID-19 pandemic, many students are more familiar with online learning and the adaptability it provides [7–10], particularly for remote locations. The online component makes the course more inclusive, potentially allowing mature learners and those from geographically disparate regions to participate in the course [11, 12]. There is a growing trend towards hybrid programmes among health professions, including physician associate (PA) education [3]. Students who live at home can benefit from family capital and social supports to help them achieve university success [13]. Other benefits can include less time spent commuting [14] and allowing students to learn among their communities where they may be employed while at the same time helping address workforce shortages in these communities [6]. In considering this project, a steering committee was formed, comprised of university and programme officials. Part of the process included carrying out a SWOT analysis in addition to a stakeholder analysis to guide the project. Opportunities highlighted by this analysis included support from consultants outside Dublin to train PAs and provide employment for them. At the time of the study, there was one university offering the Physician Associate Studies programme in Ireland. Following a successful pilot project with the Irish Department of Health, the university has invested substantially in the programme.

Giving access to students from distinctly different backgrounds can add to the diversity of the student group [15]. Students can also benefit from their community networks as they enter a new career [13]. Internet challenges (adequate broadband coverage) in some parts of Ireland have been acknowledged during the recent pandemic and were seen as a barrier to effective remote work or educational experiences. It has been reported that one in six higher education students in Ireland come from areas experiencing poor broadband coverage, with large differences by geography [16]. However, decentralisation of programmes can attract graduates from remote areas of a country, who can be more favourable to employment in their local areas [3, 17]. It is envisaged that training students in local clinical settings can help build up a community of PAs for primary and secondary settings, which currently have challenges in attracting healthcare professionals, including doctors, to vacant posts. However, designing a hybrid programme is not without its challenges. Blending face-to-face with synchronous online teaching requires careful planning and engagement from faculty and students alike. While the ultimate goal is to create an inclusive learning environment, it cannot be assumed that this is the case. The overall aim of the study was to evaluate the hybrid model of the PA programme, from the perspectives of students and their teachers.

## Methods

**Fig. 1:**
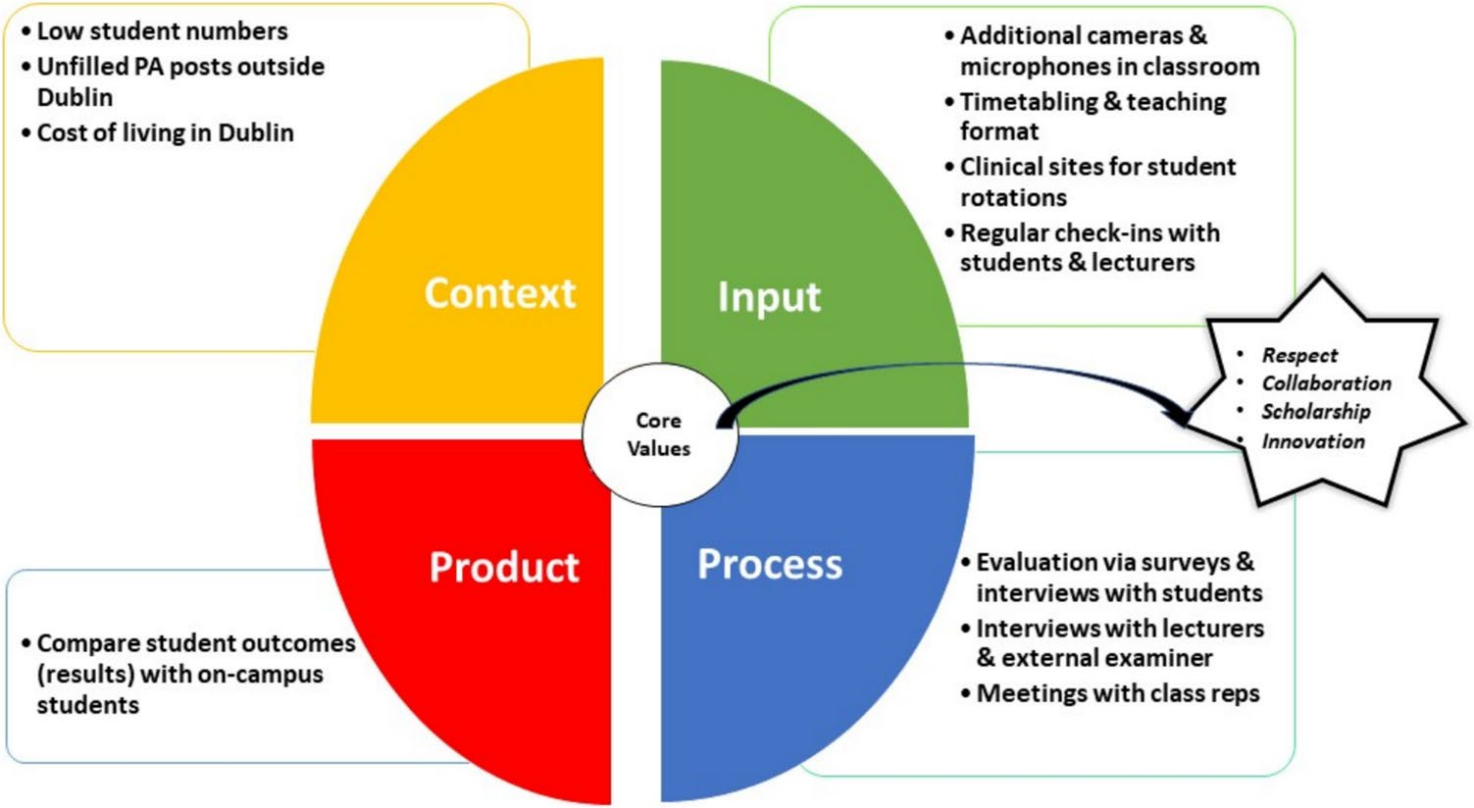
CIPP model

This was a mixed methods study, using surveys, focus groups, and one-to-one interviews as well as document analysis of programme board meetings. Ethics approval was obtained from the organisation to carry out the study. The Context, Input, Process, Product (CIPP) model (Fig. 1) that provided a framework to establish the effectiveness and/or success of the hybrid model guided the evaluation of this initiative [18]. The CIPP model particularly focuses on the inputs and process of an innovation. To satisfy the Input part of the CIPP model, additional cameras and microphones were set up in the classroom, timetabling was adjusted to facilitate twice-monthly on-campus days for hybrid students, and communication with students, lecturers, and clinical staff was ongoing. The Process section of this evaluation is focused on mixed methods data collection via interviews and surveys from students, lecturers, and the external examiner. Data from programme board meetings with class representatives was also included here.

All students who were part of the hybrid model in 2024 were surveyed in addition to the standard end-of-semester evaluations via the organisation’s Quality Enhancement Office (QEO). The questions in the survey were extracted from a larger end-of-semester survey which had been validated by the QEO in the organisation. Data analysis was carried out by Survey Monkey. In addition to surveys, focus group meetings were set up to collect data from students, lecturers, and clinical staff about their experiences of the hybrid programme format. Finally, students who progressed into year two of the 2023 hybrid programme were also invited to a focus group interview. Students were asked four questions (Table 1), based on items raised in the literature reviewed on hybrid programmes. Year two students were asked an additional question about clinical placements, as they were full-time in clinical sites during this year. Interviews were carried out by a researcher outside the department, who did not know the students and was not involved in their education. Questions asked by lecturers and the external examiner are included in Table 1. Thematic analysis was carried out for interview data. Minutes of programme board meetings, held once each semester, with class representatives, faculty, module leaders, and programme coordinator were analysed by themes too. These meetings served as ongoing feedback opportunities. Action points were noted and included with themes from interviews.

**Table 1:** Interview questions

Students	Lecturers	Clinical Staff	External examiner
How do you feel you have integrated with other students in the class?	Tell me about your experience of teaching on the programme since the introduction of the hybrid model?	Were you aware of this Physician Associate Studies programme before having these students on clinical rotation?	Have you had experience with hybrid Physician Associate Studies’ programme before working with the RCSI programme?
Are there any classroom activities you did not feel part of when attending online lectures?	Did you have previous experience of this style of teaching?	Is there anything RCSI could do to support you further in teaching/supervising students on the hybrid model of the programme?	In your experience to date with the programme is there anything you would recommend to develop this model further?
Have you had previous experience of online learning?	Do you have any suggestions for how class integration can be enhanced?	Would you be prepared to take more Physician Associate Studies’ students in the future?	Have you noticed any difference to date in student performance between the on-campus and hybrid students?
Do you have any suggestions for how class integration can be enhanced when attending online classes?	Have you noticed any difference to date in student performance between the on-campus and hybrid students?	Have you noticed any difference to date in student performance between the on-campus and hybrid students?	Would you recommend this model of teaching for other Physician Associate Studies’ programmes?
What has been your experience of your clinical rotations outside of Dublin? (Year 2)			

Students from year one had the option to tick a box at the end of their survey, if interested in taking part in an interview. Year two students received an invite from the gatekeeper of the study. Clinical teachers and lecturers also took part in focus groups. A research assistant who is not a member of the faculty and does not have any involvement in teaching on the programme carried out the interviews with students. The Product component of the CIPP, which relates, to the student results and successes on the programme was compared to those of students, from previous years and to students attending on-campus in the current year.

## Results

### Surveys

All students (*n* = 15) in year one attending the hybrid model of the programme completed a survey which investigated their attitude to online learning and their engagement with the programme. One of the hybrid students took a leave of absence leaving 15 students, eligible to be included. There was an 80% response rate to the survey. Table 2 summarises their level of agreement with the statements presented to them.

**Table 2:** Attitudes to online learning and programme engagement

Statement		Agree/strongly agree (%)	Neutral (%)	Disagree/strongly disagree (%)
1	Live online classes included polls and/or questions that encouraged me to engage	83	8	8
2	I had an opportunity to ask questions during live online classes	83	17	0
3	Small group online classes/tutorials included breakout rooms to encourage peer working	83	17	0
4	Online Moodle content (recordings and other materials) increased my understanding of the module	67	8	25
5	Teaching materials /information were made available on time (via Moodle or otherwise)	75	25	0
6	Sufficient technical support and training was available	58	25	17
7	I feel connected to the other class members	50	33	17
8	The on-site sessions keep me engaged so that I feel a sense of belonging to the whole group	92	0	8
9	The twice-monthly on-site classes are an important part of my learning	83	0	17
10	I would recommend the hybrid option of this course to a fellow student	92	0	8

While the majority of statements received agreement or strong agreement, it is noteworthy to highlight that only half the sample felt connected to other class members. Thirty-three percent of the sample remained neutral on this statement, and 17% disagreed. Another statement which received a 58% agreement was the amount of technical support and training received. Seventeen percent disagreed with the statement, while 25% of the sample remained neutral.

### Interviews

Two separate focus group interviews were carried out, i.e. six of the first-year group (*n* = 15) and five of the second-year group (*n* = 5) who volunteered to be interviewed. All hybrid students were invited to take part in these interviews. Thematic analysis was carried out with four main themes emerging across interviews. Themes which emerged from the questions asked were as follows: previous online learning experience, social isolation, creating an inclusive environment, and student performance.

#### Previous online learning experience

Most of the students had experienced online learning for their undergraduate degree. The lecturers interviewed had all delivered programme content during the pandemic and the learning gained from that experience. To encourage engagement, one lecturer experimented with games for classroom activities, using breakout rooms. It was still challenging for lecturers if they had not met students face-to-face. A suggestion of meeting the group during induction week and having a copy of their photos and names beside the desktop was recommended. One student in year two admitted that.

…the biggest issue for me was proctor issues. I was 40 mins late starting one exam and was very upset. I didn’t know that others also had problems. We need to know that it is not just us. They should communicate that to us. (Student Y2 C)

The ‘not knowing’ caused them anxiety which they suggested could have been alleviated if faculty let them know there was an overall connectivity or access problem. As per survey results (Table 2), only half the sample felt connected to other class members.

#### Social isolation

Not being present in the classroom caused one student to admit to feeling isolated at the beginning of the programme:

At the start, I felt isolated not by the primary lecturers but the guest ones…not aware of us. (Student Y2 D).

Other students agreed that some lecturers did not seem to be aware of the students online. One of the lecturers did agree that, coming out of teaching in the pandemic, they were more familiar with students accessing the lectures after the event while the PA students were required to attend the lectures synchronously. Lecturer A admitted that.

The issue with trying to engage the students online is the delay in waiting for them to text or for them to turn on their cameras. You are trying to cover a lot of content so any delays mean that you will not get through the slides. (Lecturer A)

The students suggested that lecturers leave on the audio and camera after the class had finished so that they could engage in the chatter with their peers between classes. While attempts were made to link ‘at home’ students with ‘on-campus’ students via scheduled peer-to-peer meetings, the ‘at home’ group set up their own WhatsApp group chat, as they felt they had more in common with each other. The feeling of isolation was exacerbated for students at home when taking their online exams.

#### Creating an inclusive environment

Data suggests that, overall, the student experiences were positive. In particular, they liked the peer-to-peer feedback sessions and the on-campus contact twice a month. The following are examples:

I feel integrated with the other students. We did these peer-to-peer meetings every second week…between a hybrid and on-campus student. That really helped get to know the other students that we might not see every day. I think that helped. (Student Y1 A)

Some recommendations for these interactions, during on-campus days, included the following:

…there could be an extension to a lunch or another break in the middle…just to give you a chance to be chatting to people in person, because you do nearly end up eating your lunch chatting to whoever's beside you. (Student Y1 F)

This data suggests that in-person contact for students is important to them. Other areas for improvement centre around guest lecturers ensuring that they treat the online students as if they were present in the classroom. The following is an example from a year two hybrid student regarding them integrating with the rest of the class:

We feel more connected to the hybrid part of the group because we have the same context - all of us studying from home... we all have lunch together when on-campus - it is a natural interaction (Student Y2 B)

Students suggested more online meetings between them and faculty while on clinical rotations in addition to on-site visits. They also recommended that more information be communicated to the clinical sites about the PA role. When students from the university attend new hospital sites, there might be delays with some logistics, including identity cards for student access and identification. In addition, the students sometimes felt that staff were not aware of their role:

There is a lack of teaching and communication about the PA role. When we went to hospitals, they didn’t know who we were… It seems they are being prepared for us but maybe this is not communicated to the right people. (Student Y2 C)

#### Student performance

There was consensus from lecturers and the external examiner that there was no difference in the hybrid students’ academic or clinical exam performance in the programme. This observation was also echoed regarding their clinical performance by clinical teachers (CT). They agreed that they only saw a difference between students, based on their backgrounds, with some students entering the programme from a science background and others having a healthcare background. One clinical teacher commented:

…the student with the clinical background definitely seems a bit more comfortable and at home in the hospital. (CT 1)

Some recommendations from clinical teachers included the need to share feedback with them on the strengths and weaknesses of students assigned to them. Comparing student outcomes (Product of CIPP model), between on-campus and hybrid students shows that the hybrid students did as well or better across both first and second years.

Since the completion of the study, the second-year group has completed the programme and has taken their national PA exam, which is an exam which allows PA graduates to register with their professional body (Irish Society of Physician Associates). Again, there has been no significant difference in the hybrid graduates’ results to the on-campus students.

## Discussion

Equivalency in all aspects of the educational experience is one of the biggest challenges for educators. Clinical partnerships with regional hospital groups require a strong commitment to PA education. Literature on satellite campuses has brought this consideration to the forefront [13, 19]. Teacher-student relationships are also a consideration because meeting students, for the most part online, could challenge the ability to build adequate rapport, but this may be specific to the particular student [15]. Despite adapting to new modes of learning, students find online learning challenging in terms of technology [20]. Where infrastructure is not optimum, this can be frustrating for the student and lecturer alike [21]. According to one study carried out during the pandemic [22], learning alone at home, lacking access to learning resources, and inaccessibility to other e-learning platforms are the top three constraints students faced during e-learning which caused them anxiety. It is the internet which affords the student the opportunity to take their course from home [20]. The experiences of hybrid learning among health sciences students have been described through different categories [23]. According to Eija et al., the competence of the educator in technology and pedagogy style can influence successful hybrid teaching. Hybrid learning support for students was also recommended in our study. Overall, the students believed that hybrid learning enabled their freedom of choice, flexibility, and accessibility of participation, as well as a variety of learning experiences [23].

In our study, a lecturer referred to not knowing which student they were talking to online as they had no face to match the name. For the most part, students used the ‘chat’ resource because turning on the camera took time and sometimes interfered with their internet connectivity, a problem found by others [24]. Some students admitted to feeling isolated at times when attending the live-streaming of lectures. Only 50% of the first-year student sample agreed to feeling connected to the class, with another 33% remaining neutral on this statement. Social isolation has been acknowledged by students and lecturers during the pandemic experience of online learning [25]. The lack of social interaction with their peers in the classroom was verbalised by students in our study too. The concern about maintaining student engagement is more controversial than ever when delivering online learning sessions. While lecture-based classes were the norm for medical education, there is now more of a move towards active participation of the student [26]. Engaging students in the classroom during synchronous education sessions requires upskilling of the lecturer as well as the student. While upskilling academic staff on the use of digital platforms occurred at a rapid pace during the pandemic, ensuring the most effective use of the platforms for student engagement during new pedagogical practices can prove challenging. As lecturers continued their didactic lecture style teaching via remote delivery, such practices may not suit long-term student engagement capable of supporting learner motivation and the development of higher-order thinking skills [27]. Many of the programmes at the study site have returned to on-campus teaching and learning; however, our programme is unique in that there is half the student group in the classroom, while the remainder is attending online during the sessions. Synchronous distance education was not significantly different from traditional education in effectiveness and had higher satisfaction ratings in one systematic review and meta-analysis [28]. In our study, a lecturer agreed that he sometimes forgot that students were attending synchronously. The students picked up on this lack of awareness of their presence. This limited interaction between students and lecturers can enhance social isolation and have a negative effect on student engagement [29]. While the use of strategies such as breakout rooms is used to encourage group work and participant engagement, a balance between the flexibility and convenience of online learning strategies and the need for in-person interaction is important, as no one-size-fits-all [30].

While lecturers try to maintain equality of the learning experience, this can prove complex for delivery when dealing with hybrid students [31]. One study identified the need for flexibility, trust, the human element, and ownership as key themes when designing hybrid programmes [32]. Flexibility referred to the tools that the teachers used to create a common learning space. Trust and the human element included the interaction and building of trust between students and their lecturers. Ownership of learning referred to learning motivation, engagement, and the ability of the learner to be self-directed. According to MacNeil et al. [33], interactivity is key to synchronous learning. Without this interactivity, lectures could be recorded and viewed at a later date. A deficiency in online learning can be the lack of theory in the design of the curriculum, and this requires faculty development [33]. Studies on the effectiveness of hybrid learning are inconclusive. One meta-analysis [34] suggests that it paves the way for students studying science and biology. Another study [35] attempted to set up a performance prediction and alert method in hybrid learning. This has proven very challenging for both online and offline students. Hefnawi et al. [36] found that dental students preferred the hybrid learning option. They assessed student performance on a problem-based learning biomedical sciences curriculum and found it to be effective for learning fundamental knowledge and developing critical thinking skills. There was no difference in overall scores of students taking in-person and hybrid learning options [36].

## Conclusion

Innovative approaches that help to grow the healthcare workforce are critical to meet the current and projected needs due to an aging population, post-COVID-19 demands for health services, and career mobility for mature learners. This study demonstrates that introducing a hybrid model does not compromise quantitative educational outcomes and student success. Qualitative findings do reinforce the need to decrease social isolation and increase the skill set of faculty to approach the delivery of hybrid education, which includes instructional design that is more creative and inclusive for the learner. Programme engagement with hybrid students should be ongoing and responsive to student needs to prevent further isolation. Building and sustaining clinical partnerships with local healthcare institutions requires time. Furthermore, the uptake of a new health professional group also requires local support that ensures a welcoming and inclusive environment. While this study is limited by its small numbers and growth in hybrid students was influenced by the availability of funding towards tuition, it does highlight some key findings, in particular, the ongoing need to create an inclusive environment for students no matter the programme delivery. Being cognisant of social isolation feelings for students needs to be at the forefront of teachers. With the anticipation of growth in university enrolments, higher education will need to adopt new approaches to teaching and learning. This can be challenging when the programme also has a clinical hands-on focus such as healthcare competencies. While much has been achieved in upskilling teachers and students in an online learning environment, there is still room for improvement when offering a hybrid education model. Including and engaging students during synchronous teaching sessions, with some students on-campus and others ‘at home’, are not easy tasks. Though great efforts can be made to help online students feel included, it is challenging to convey university culture without experiencing the campus itself. The in-person component of hybrid education gives students a chance to experience campus and university culture. Competing demands for clinical staff include the increase in the number of medical students in training and their unfamiliarity with the PA role. With clinical hours making up a majority of the programme, work needs to continue in order to make the learning seamless for hybrid students.

## Data Availability

All relevant data is part of the manuscript. More data will be made available upon request.
